# FusionDiff: a dual-path diffusion-based framework for few-shot authenticity analysis of ceramic microstructures

**DOI:** 10.1038/s41598-026-57035-x

**Published:** 2026-06-13

**Authors:** Wenxuan Fu, Xing Xu, Yuanhui Huang, Xiaotong Li, Xuewen Xia, Yinglong Zhang

**Affiliations:** 1 School of Physics and Information Engineering, Minnan Normal University, Zhangzhou, 363000 Fujian China; 2 Center for China-ASEAN Regional Collaborative Development, Minnan Normal University, Zhangzhou, 363000 Fujian China; 3 School of Computer Engineering and Science, Shanghai University, Shanghai, 200000 China

**Keywords:** Microscopic ceramic structures, Few-shot learning, Diffusion models, Dual-path fusion, Engineering, Materials science, Mathematics and computing

## Abstract

The authenticity of ceramic components is closely tied to their microscopic structures, making automatic and accurate identification essential for quality control. However, this task is often constrained by the scarcity of labeled samples. This study investigates the potential of large-scale pretrained diffusion models as feature extractors, leveraging the rich visual priors embedded in their generative processes to provide a robust semantic foundation for small-sample learning. To address the limitations of the original U-Net in global representation modeling and the weak local-detail sensitivity of DeiT, we propose a dual-path fusion encoder, FusionDiff. Within a frozen Stable Diffusion V1.4 framework, CNN and adapter-enhanced DeiT paths operate in parallel and are deeply integrated via feature gating. Following a “self-supervised pretraining + supervised fine-tuning” paradigm, classification is performed using a Random Forest classifier. On our custom ceramic dataset, FusionDiff achieves a test accuracy of 99.07%, outperforming SD-CNN (97.44%), DeiT (96.30%), and ResNet50 (97.00%) under a unified self-supervised evaluation protocol. Even under extremely small-sample conditions (*n = 50*), the model attains 90.7% validation accuracy, demonstrating competitive data efficiency and cross-domain generalization capability.

## Introduction

High-end ceramic products—ranging from advanced structural components and functional electronic ceramics to antique artistic porcelain—derive their significant value from a synergy of intrinsic performance and authenticity. These attributes are fundamentally encoded within their microscopic structures^1,2^. Historically, authenticity verification has relied on expert-led visual inspections of macroscopic features such as vessel morphology, glaze tonality, and decorative motifs. However, the recent proliferation of high-fidelity generative models, particularly Diffusion and Generative Adversarial Networks (GANs)^3,4^, has triggered a technological “arms race”. Counterfeiters can now synthesize increasingly deceptive images that bypass traditional inspection, posing unprecedented challenges to cultural heritage preservation and industrial quality control^6^.

In contrast to macroscopic aesthetics, which can be superficially imitated, ceramic microstructures are the products of complex, stochastic physicochemical processes. The spatial arrangement of grain boundaries, pores, and glass-phase distributions constitutes a unique “microstructural fingerprint”. While high-magnification optical or electron microscopy provides rich discriminative cues, the practical application of deep learning in this domain faces a dual bottleneck. First, labeled microstructural data for rare materials or archaeological artifacts are inherently scarce, leading to a few-shot learning (FSL) scenario^7^. Second, while synthetic micrographs may achieve visual verisimilitude, they often lack the subtle structural irregularities and multi-scale non-uniformities characteristic of real-world sintering kinetics.

Current FSL paradigms, primarily based on meta-learning^8^ or metric-learning^9,10^, have shown promise in industrial inspection. However, their performance often plateaus when confronted with fine-grained textures under extremely limited supervision. A burgeoning direction involves leveraging large-scale pretrained diffusion models as universal feature extractors^11,12^. Unlike purely discriminative CNNs^13^ or Transformers^14,15^, diffusion networks implicitly internalize rich generative visual priors^16^ that capture intricate morphologic and structural relationships. Nevertheless, a critical gap remains: the standard diffusion U-Net architecture is inherently biased toward local texture modeling, often overlooking the global contextual semantics that are vital for authenticity analysis.

To bridge this gap, we propose FusionDiff, a dual-path fusion framework anchored on a frozen Stable Diffusion backbone^5^. Our approach synergistically integrates a CNN pathway (derived from the Stable Diffusion U-Net encoder) with a lightweight, adapter-enhanced DeiT Transformer pathway^15^. By employing a gated fusion module^17^, FusionDiff adaptively calibrates local texture sensitivity with global structural context, yielding a more holistic representation for microstructural discrimination.

The primary contributions of this work are three-fold:

- We propose a novel framework that repurposes generative diffusion priors for the few-shot authenticity analysis of ceramic microstructures, providing an effective approach to distinguishing real ceramic micrographs from AI-generated counterfeits.
- We design a dual-path encoding architecture that harmonizes local-scale diffusion features with global-scale Transformer semantics, coupled with a gated mechanism to ensure stable feature integration under data-scarce conditions.
- Extensive evaluations on the Ceramic Microstructure Dataset (CMD) show that FusionDiff delivers the best overall performance at larger training scales while maintaining competitive accuracy in extremely low-data settings (*n ≤ 50*).

## Results

Table 1 reports the classification performance of different models on the CMD dataset, where an approximately class-balanced training subset of 1071 images was used. To ensure a fair comparison, all models adopt a unified evaluation protocol in which learned feature representations are used to train a Random Forest classifier for final classification. The training protocols for each model are as follows. FusionDiff employs a frozen Stable Diffusion backbone with a dual-path architecture, trained via diffusion-based pretraining followed by contrastive self-supervised learning on CMD. SD-CNN^5^ uses the same frozen Stable Diffusion encoder as a single-path baseline, trained with the same contrastive self-supervised objective. DeiT^15^ is initialized with Stable Diffusion encoder weights and fine-tuned using a contrastive self-supervised objective on CMD. For all remaining baselines, including ResNet50^13^, DenseNet121^18^, Swin-Tiny^19^, CLIP-ResNet50^20^, ViT-L/16^14^, and CNN-LSTM^21^, the models are initialized from their original pretrained weights and fine-tuned on CMD using SimCLR^22^ self-supervised learning, followed by Random Forest classification.

FusionDiff achieves the highest test accuracy among all compared methods, reaching 99.07%. Under this unified evaluation protocol, FusionDiff consistently outperforms all baselines, demonstrating that the dual-path architecture itself contributes to improved representations beyond the benefit of pretraining alone. Notably, the performance gap between FusionDiff and the strongest baseline is modest, which reflects the effectiveness of the unified self-supervised protocol in reducing the influence of training procedure differences.

Compared with the standalone SD-CNN encoder and the DeiT Transformer, the proposed dual-path architecture consistently improves performance. These results indicate that combining local texture sensitivity from diffusion features with global contextual modeling from Transformers leads to more discriminative representations for ceramic authenticity analysis.

**Table 1:** Performance comparison of different models.

Model	Test accuracy	Validation accuracy	Fake F1	Real F1
FusionDiff (ours)	99.07%	98.95%	0.99	0.99
SD-CNN (baseline)	97.44%	96.96%	0.97	0.97
CNN-LSTM	97.50%	98.80%	0.97	0.99
DeiT	96.30%	96.70%	0.96	0.98
ResNet50	97.00%	98.80%	0.97	0.97
DenseNet121	97.50%	97.47%	0.97	0.98
Swin-Tiny	97.60%	97.27%	0.97	0.99
CLIP-ResNet50	96.50%	98.94%	0.96	0.97
ViT-L/16	90.50%	90.70%	0.90	0.91

### Few-shot data efficiency

To evaluate robustness under limited supervision, we conduct few-shot experiments on CMD using class-balanced training subsets containing 50, 100, 200, 500, and 1000 labeled samples. These settings are separate from the standard 1071-sample training configuration used in Table 1. To ensure a fair comparison, all baselines follow the same unified self-supervised pretraining and Random Forest classification protocol as described in “Results”.

As shown in Table 2, FusionDiff remains competitive under extremely limited supervision and achieves the highest validation accuracy at *n=1000* (98.9%), which is not reached by any single-path baseline. Although several baselines perform similarly at *n=50*, FusionDiff shows a steady improvement as the number of labeled samples increases, indicating that the complementary CNN and Transformer pathways become increasingly effective with more supervision.

**Table 2:** Sample size sensitivity: validation accuracy (%) after row–column transposition.

Model	50	100	200	500	1000
FusionDiff	90.7	91.2	94.9	96.0	**98.9**
SD-CNN	85.6	89.8	89.5	95.9	97.0
DeiT	86.6	91.3	93.2	95.4	96.0
ResNet50	90.0	92.0	93.2	95.1	96.4
DenseNet121	91.0	92.5	94.8	96.2	97.5
Swin-Tiny	90.8	92.2	94.4	96.3	97.3
CLIP-ResNet50	90.0	92.0	93.2	95.1	96.4
ViT-L/16	85.9	87.4	89.0	88.8	90.3
CNN-LSTM	90.2	91.8	92.3	95.5	97.1

### Fusion strategy analysis

To evaluate the effectiveness of the proposed gated fusion mechanism, we compare several alternative feature fusion strategies, including simple concatenation, weighted summation, and self-attention fusion.

**Fig. 1:**
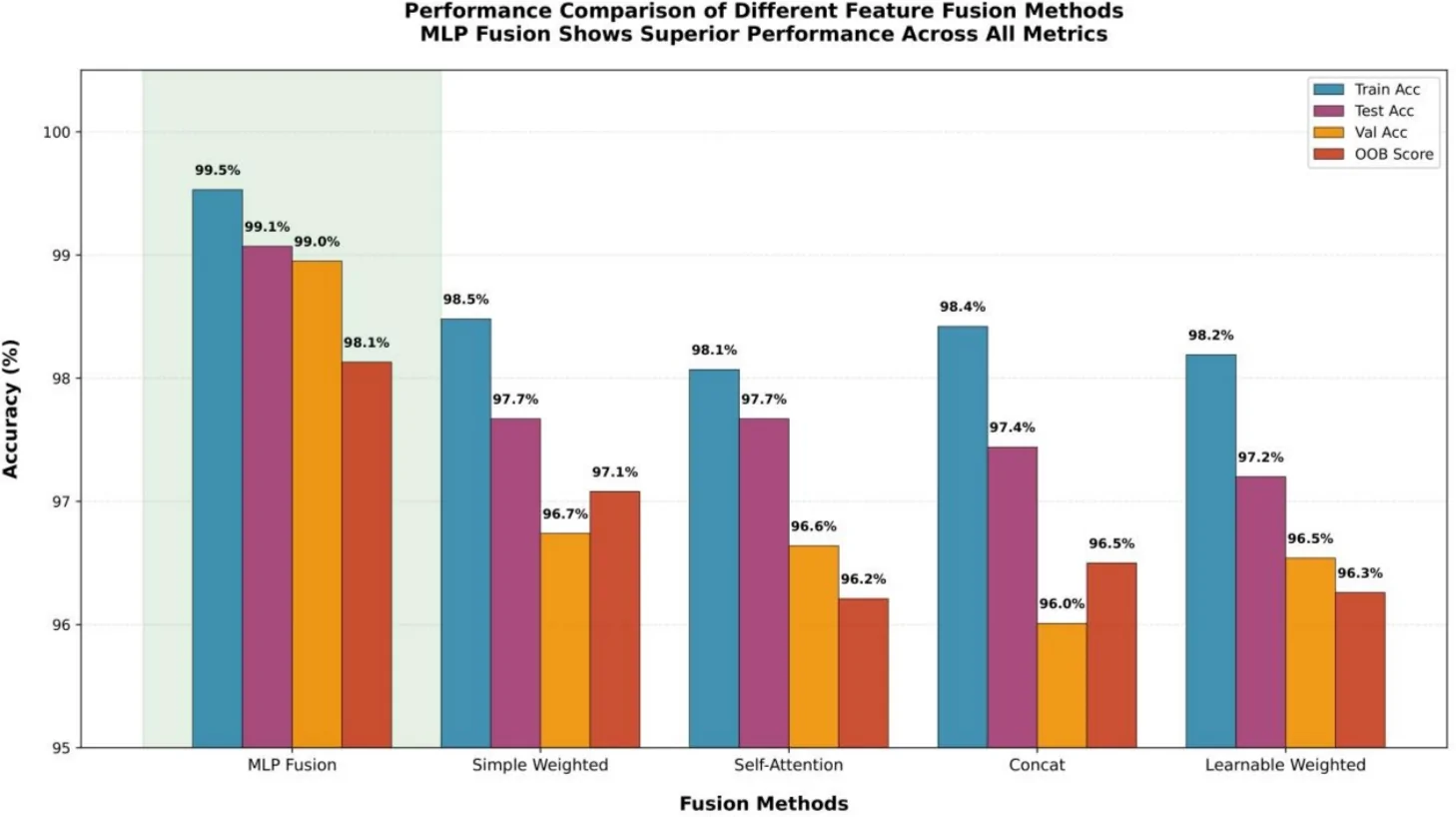
Performance comparison of five feature fusion strategies.

As shown in Fig. 1, the proposed MLP fusion module achieves the highest performance among the evaluated fusion strategies. In contrast, naive concatenation and fixed-weight fusion lead to lower accuracy, indicating limited ability to balance local and global features.

These results suggest that adaptive gating enables more effective integration of CNN and Transformer representations, allowing the model to dynamically adjust their relative contributions.

### Activation map analysis

**Fig. 2:**
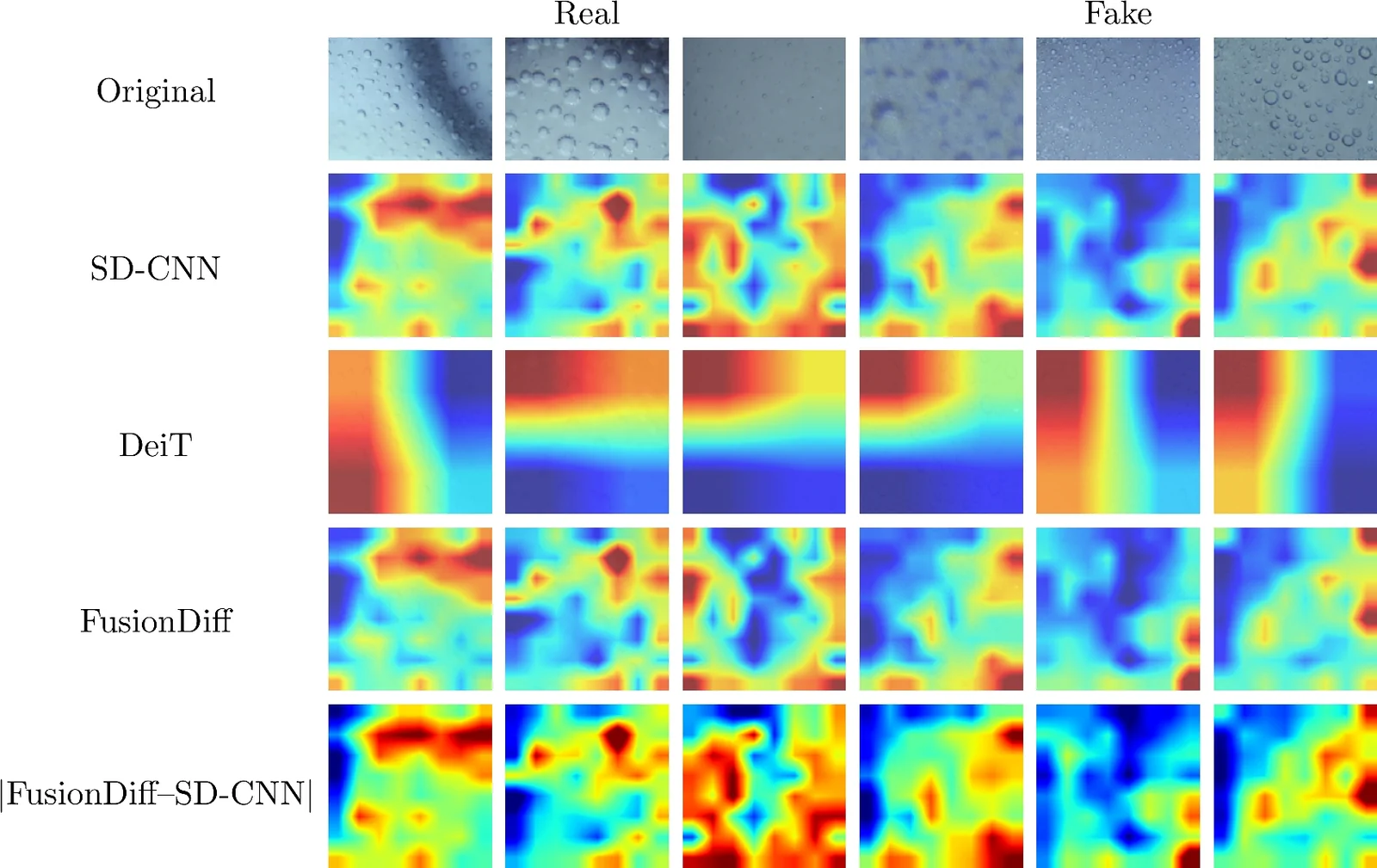
Comparison of real/fake samples and feature response maps across SD-CNN, DeiT, and FusionDiff (left three columns: real samples; right three columns: StyleGAN2-generated fake samples). All heatmaps share a unified jet colormap normalized to [0, 1]. The last row visualizes the absolute feature-level difference between FusionDiff and SD-CNN.

To further investigate the learned feature representations, we visualize the feature activation maps produced by different models. For SD-CNN, activations are extracted from the final encoder layer; for DeiT, from the last Transformer feature output; and for FusionDiff, from the hook-based gated fusion output, which integrates CNN and aligned Transformer features via adaptive gating. As shown in Fig. 2, the SD-CNN branch mainly focuses on fine-grained local texture patterns, while the DeiT branch captures broader structural context. FusionDiff produces activation patterns that reflect contributions from both pathways, yielding more stable and semantically consistent responses. The relatively small magnitude of the feature-level difference between FusionDiff and SD-CNN reflects the effectiveness of the feature adaptation module, which aligns DeiT representations towards the CNN feature space prior to fusion. The remaining modulation highlights structurally informative regions where the Transformer pathway provides complementary refinement beyond the CNN encoder alone.

### Cross-domain generalization

**Fig. 3:**
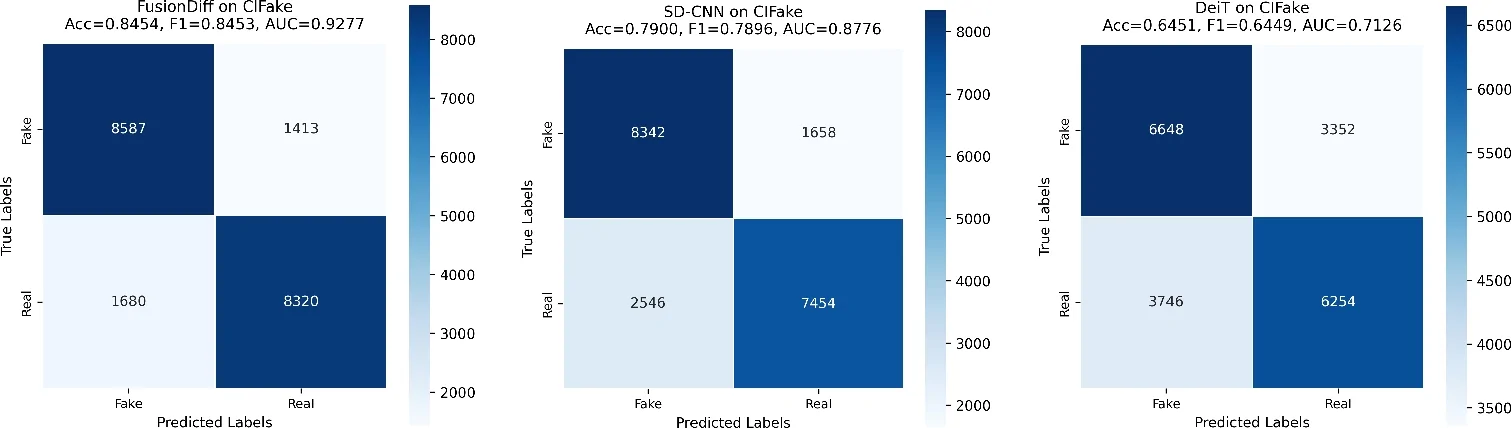
Confusion matrix comparison of FusionDiff, SD-CNN, and DeiT on the CIFake dataset. Quantitative metrics are reported above each confusion matrix.

To evaluate the generalization ability of the proposed model, we further tested FusionDiff on the CIFake^23^ dataset. As shown in Fig. 3, FusionDiff achieves an accuracy of 84.54%, macro F1-score of 0.845, and AUC of 0.928 on the CIFake dataset, outperforming SD-CNN (Acc = 79.00%, F1 = 0.790, AUC = 0.878) and DeiT (Acc = 64.50%, F1 = 0.645, AUC = 0.713). This result suggests that diffusion-based representations capture transferable structural patterns across different image domains, and that the dual-path fusion strategy contributes to more robust cross-domain generalization compared with single-path baselines.

### UMAP embedding analysis

**Fig. 4:**
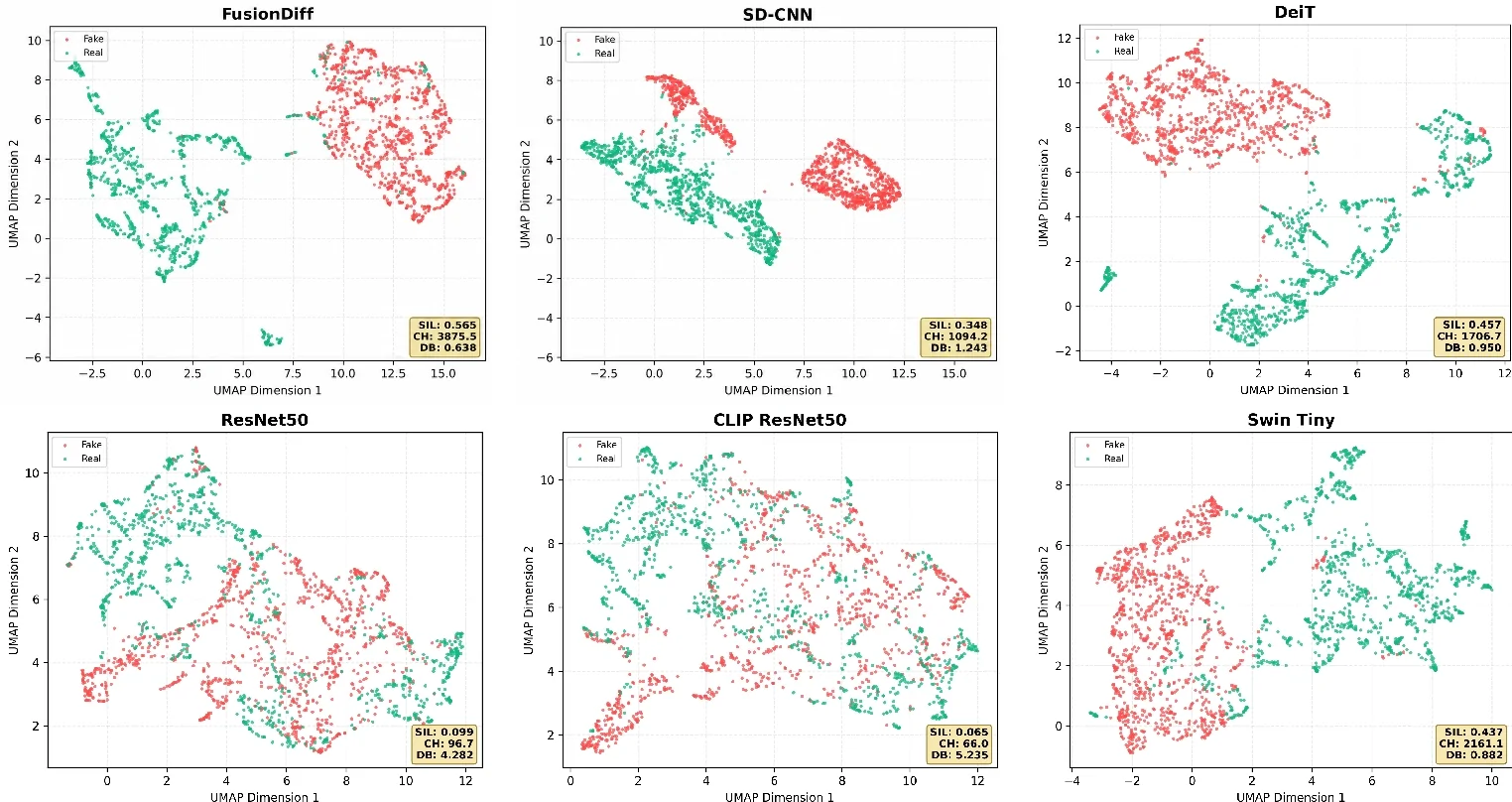
UMAP-based comparison of feature embedding quality across models. Each subplot reports the Silhouette score (SIL), Calinski–Harabasz index (CH), and Davies–Bouldin index (DB).

To further examine the discriminative quality of learned representations, UMAP projections of high-dimensional embeddings from six models are visualized in Fig. 4. FusionDiff produces the most compact intra-class clusters and the clearest inter-class separation, with real and fake samples forming two well-defined regions. This is further confirmed by quantitative metrics: FusionDiff achieves the highest Silhouette score (0.565) and Calinski–Harabasz (CH) index (3875.5)—both indicating better cluster separation and compactness—and the lowest Davies–Bouldin (DB) index (0.638), which reflects minimal intra-class scatter relative to inter-class distance, among all compared methods. In contrast, SD-CNN shows weaker intra-class compactness with a lower Silhouette score (0.348) and higher DB index (1.243), despite achieving visible inter-class separation. ResNet50 and CLIP-ResNet50 exhibit heavily overlapping distributions with near-zero Silhouette scores (0.099 and 0.065, respectively), while DeiT shows fragmented cluster structures. These results indicate that the dual-path fusion strategy yields more discriminative and stable feature embeddings compared with single-path baselines.

## Discussion

The experimental results demonstrate that diffusion-based feature representations offer competitive advantages for ceramic micrograph authenticity analysis, with FusionDiff consistently outperforming both CNN and Transformer baselines across varying data conditions. Compared with conventional CNN and Transformer baselines, FusionDiff achieves superior classification accuracy and more discriminative feature embeddings, validating the effectiveness of leveraging generative visual priors from large-scale pretrained diffusion models.

A key factor behind this improvement lies in the complementary design of the dual-path architecture. The frozen Stable Diffusion U-Net encoder excels at capturing fine-grained local textures—such as grain boundaries, pores, and microstructural irregularities—which are precisely the discriminative cues that distinguish real ceramic surfaces from synthetic counterparts. Meanwhile, the lightweight DeiT branch handles global contextual modeling, capturing long-range spatial dependencies that purely convolutional architectures struggle to represent. The gated fusion mechanism dynamically balances the contributions of the two pathways, yielding a unified representation that attends to both local detail and global structure.

Another notable strength of FusionDiff is its data efficiency. FusionDiff remains highly competitive under extremely limited supervision, achieving performance comparable to the best-performing baselines at *n=50*, while demonstrating a consistently improving trend as labeled samples increase. This steady growth culminates in the highest validation accuracy of 98.9% at *n=1000*, a performance level that no single-path baseline reaches, underscoring the progressive advantage of the dual-path design under increasing supervision. Notably, repeated experiments reveal that the single-path DeiT baseline exhibits a mean validation accuracy of 86.6% at *n=50* (std *= 0.9*), which is consistently lower than FusionDiff’s 90.7%, suggesting that the dual-path design provides more stable representations even in low-data regimes. Furthermore, both the diffusion-model priors and the Transformer-based global modeling jointly contribute to this stability, as the two pathways maintain nearly equal contribution ratios throughout inference, indicating that neither pathway dominates and that their complementary interaction underlies the observed robustness.

The UMAP visualizations further corroborate these findings: compared with other models, FusionDiff produces more compact intra-class clusters and clearer inter-class separation, indicating that the proposed fusion strategy not only improves classification accuracy but consistently enhances the discriminability of the learned feature space.

Nevertheless, this study has several limitations. First, the current framework relies on a pretrained Stable Diffusion backbone, which introduces non-negligible computational overhead during feature extraction. Second, although the dataset covers both real and synthetic ceramic micrographs, the diversity of material types and imaging conditions remains limited. Future work will focus on developing more lightweight fusion architectures and extending the framework to a broader range of materials and industrial inspection scenarios. Exploring self-supervised or domain-adaptive training strategies may further improve the robustness and scalability of diffusion-based feature representations.

## Methods

### Model architecture

**Fig. 5:**
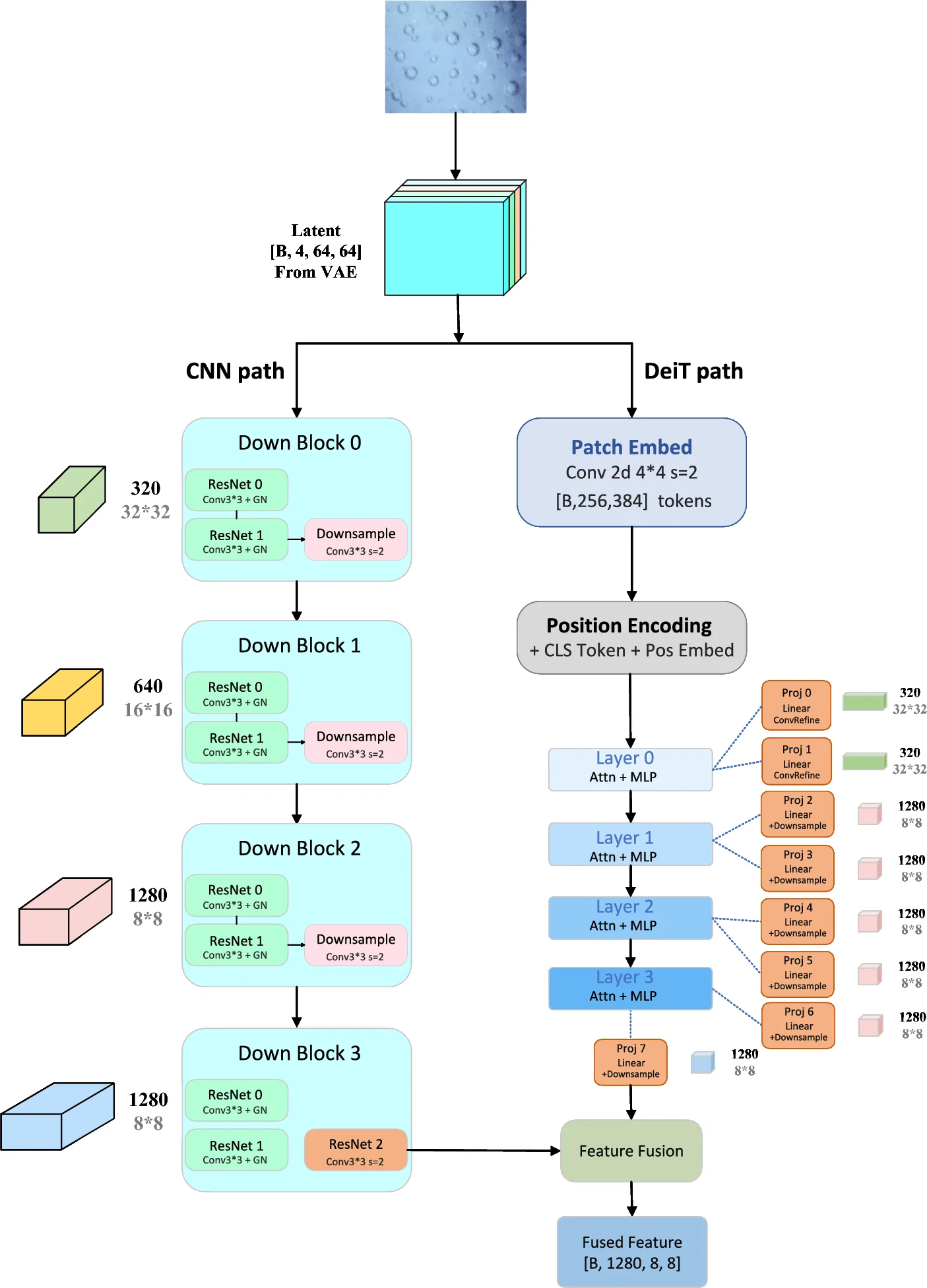
Overall architecture of FusionDiff.

FusionDiff was developed within the Stable Diffusion v1.4 framework as a hook-based dual-path fusion U-Net (HookBasedDualPathUNet). The model was designed to enable feature interaction between a convolutional branch and a Transformer branch while preserving the pretrained diffusion backbone. As shown in Fig. 5, the input image was first encoded into a latent representation using a variational autoencoder (VAE^24^). The latent representation was then processed by two parallel pathways: the original Stable Diffusion U-Net encoder, which served as the convolutional pathway, and a lightweight DeiT encoder, which served as the Transformer pathway. Through a hook-based fusion mechanism, features from the Transformer pathway were injected into selected layers of the convolutional pathway. During training, the model predicted noise in the latent space, and the resulting latent features were used for downstream real–fake classification.

### Dual-path feature encoding

FusionDiff used a dual-path encoding strategy to extract complementary representations from the input image. The convolutional pathway was based on the pretrained encoder of Stable Diffusion v1.4 and was used to capture multi-scale local texture and spatial structure information. Owing to the large-scale text–image pretraining of Stable Diffusion, this encoder provided a strong prior representation. To preserve these pretrained representations under limited-data conditions, the convolutional backbone remained frozen during training.

**Fig. 6:**
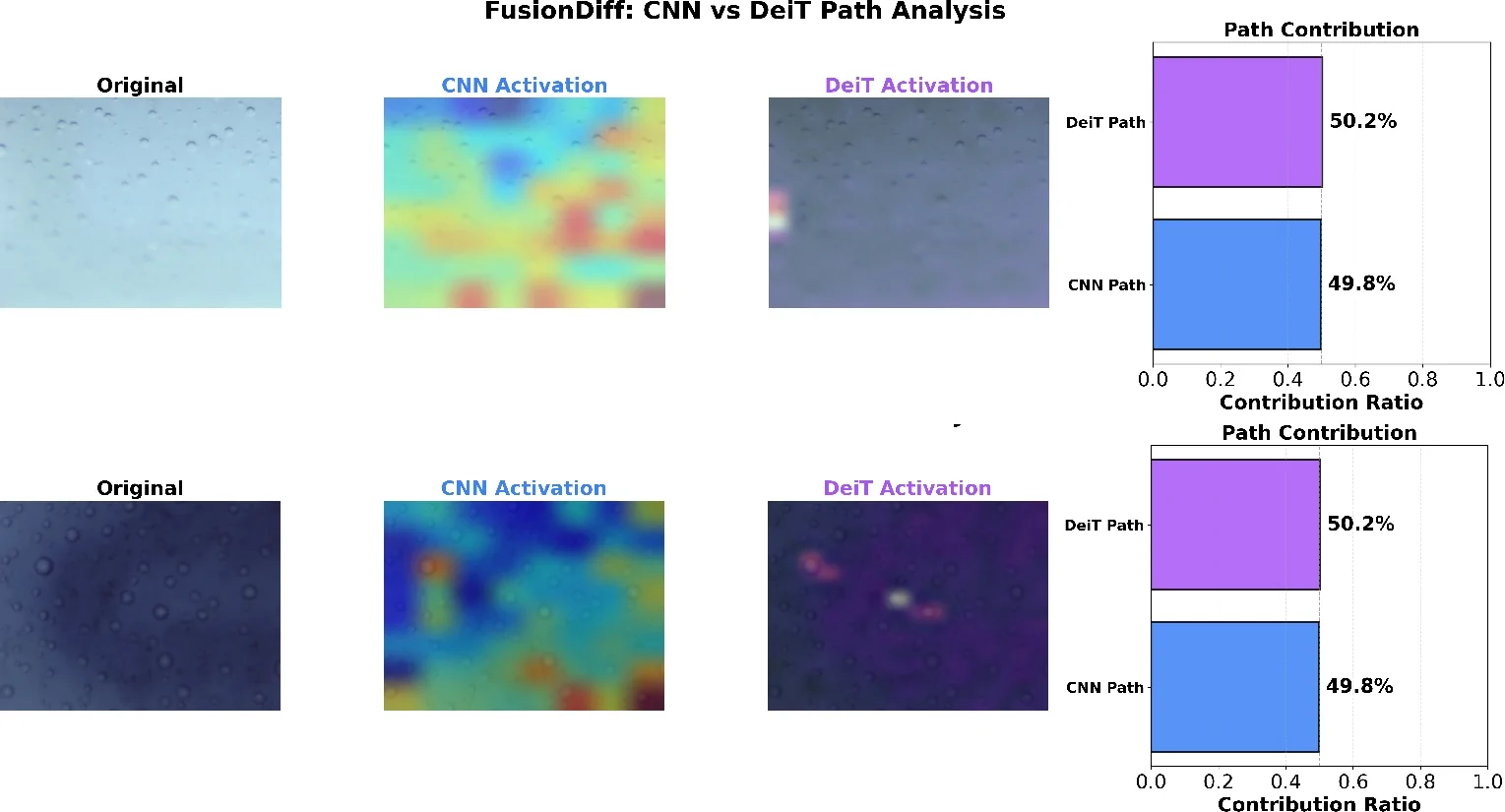
Visualization of the contributions of the CNN and DeiT pathways in FusionDiff. From left to right: original image, CNN activation map, DeiT activation map, and pathway contribution ratio.

In parallel, a lightweight four-layer DeiT encoder was used to model global contextual dependencies and long-range interactions. A shallow Transformer architecture was adopted to reduce the number of trainable parameters and to limit overfitting in few-shot settings. The outputs of the convolutional and Transformer pathways were subsequently combined through the fusion module described below. As illustrated in Fig. 6, the CNN pathway focuses primarily on local texture patterns while the DeiT pathway attends to broader structural regions, and the two pathways maintain nearly equal contribution ratios (CNN: 49.8%, DeiT: 50.2%), indicating that neither pathway dominates and that both contribute complementarily to the final representation.

### Feature adaptation and alignment

Before feature fusion, a lightweight adaptation module was applied to reduce discrepancies between the convolutional and Transformer representations. First, an affine transformation was used to calibrate the distribution of the DeiT features. Second, a *1× 1* convolution was used to match the channel dimension of the Transformer features to that of the convolutional features. Third, spatial interpolation was applied to ensure consistent feature-map resolution between the two pathways. These operations produced aligned Transformer features for subsequent fusion.

### Hook-based gated fusion

To keep the fusion mechanism lightweight and stable under few-shot training, feature interaction was introduced only at the final stage of the two pathways rather than at every intermediate layer. This choice was motivated by two considerations. First, repeated cross-layer fusion would substantially increase computational overhead and optimization complexity. Second, the final-stage features are more semantically compact and therefore better suited for aligning the local texture cues from the diffusion encoder with the global contextual cues from the Transformer branch. For these reasons, we adopted single-stage high-level fusion in the final model.

**Fig. 7:**
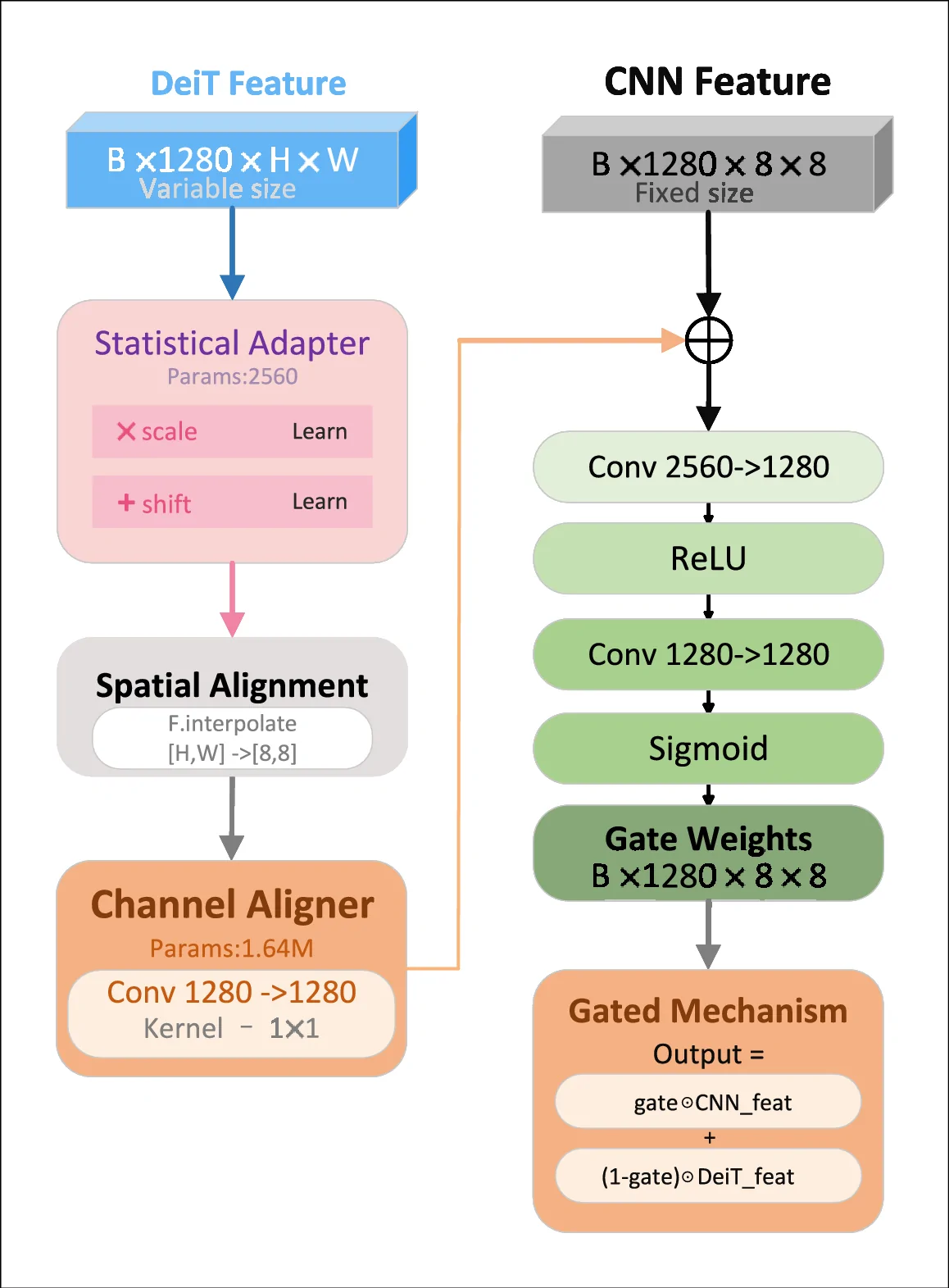
Hook-based gated fusion module integrating CNN and DeiT features with statistical adaptation and adaptive gating.

To incorporate Transformer features without modifying the Stable Diffusion U-Net architecture, a hook-based gated fusion module was designed, as illustrated in Fig. 7. A runtime hook was registered at the final encoder layer to extract the convolutional feature map $F_{\text {CNN}} \in \mathbb {R}^{B \times 1280 \times 8 \times 8}$. The aligned Transformer features $F'_{\text {DeiT}}$ were then injected through the gated fusion module, and the output was defined as

1 $$\begin{aligned} H = \Psi \!\left( \text {Fuse}\!\left( F_{\text {CNN}}, F'_{\text {DeiT}}\right) \right) , \end{aligned}$$

where *Ψ* denotes the original computation of the U-Net at the fusion layer. Because fusion was implemented through runtime hooks, the pretrained diffusion backbone required no structural modification.

#### Gated fusion module

The DeiT feature map $F_{\text {DeiT}} \in \mathbb {R}^{B \times 1280 \times H \times W}$ was first calibrated by a statistical adapter with learnable scale and shift parameters (2560 parameters in total):

2 $$\begin{aligned} \hat{F}_{\text {DeiT}} = s \odot F_{\text {DeiT}} + t. \end{aligned}$$

The calibrated features were then spatially aligned to the target resolution via bilinear interpolation (*[H,W] → [8,8]*) and projected to the target channel dimension through a *1× 1* convolutional channel aligner (1.64M parameters):

3 $$\begin{aligned} F'_{\text {DeiT}} = \text {Conv}_{1\times 1} \left( \text {Interpolate}(\hat{F}_{\text {DeiT}})\right) . \end{aligned}$$

The aligned features were concatenated with $F_{\text {CNN}}$ and passed through a two-layer gating network (2560*→*1280 with ReLU, followed by 1280*→*1280 with Sigmoid) to produce spatially adaptive gate weights $G \in \mathbb {R}^{B \times 1280 \times 8 \times 8}$:

4 $$\begin{aligned} G = \sigma \!\left( \text {Conv}_{1280}\!\left( \text {ReLU} \left( \text {Conv}_{2560} \left( [F_{\text {CNN}}, F'_{\text {DeiT}}]\right) \right) \right) \right) . \end{aligned}$$

The fused representation was then computed as

5 $$\begin{aligned} F_{\text {fused}} = G \odot F_{\text {CNN}} + (1-G) \odot F'_{\text {DeiT}}, \end{aligned}$$

where *G ∈ [0,1]* controls the relative contribution of each pathway at every spatial location.

### Training pipeline and implementation details

Stage 1: Diffusion-based pretraining. The model was pretrained on ceramic micrographs using a diffusion-based objective. A Stable Diffusion v1.4 backbone was adopted, with LoRA^25^ adapters (rank 8, scaling factor 16) injected into the U-Net attention layers for parameter-efficient adaptation. Input images were resized to *512 × 512* and normalized to *[-1, 1]*. The diffusion branch was optimized using AdamW with a learning rate of $1\times 10^{-5}$ for 15 epochs under bfloat16 mixed-precision training, with a cosine learning-rate schedule and early stopping (patience = 3).

Stage 2: Dual-path feature extraction and classification. After pretraining, only the encoder-side representation pathway of the U-Net was retained for downstream classification. A DeiT branch was introduced to provide complementary global representations, with fusion applied at the output of the last ResNet unit in the final down block of the U-Net encoder. For self-supervised representation learning, a contrastive training stage was implemented using an InfoNCE objective (temperature = 0.07) for 40 epochs, with AdamW (learning rate $5\times 10^{-5}$, weight decay $1\times 10^{-4}$) and a cosine annealing schedule.

Downstream classifier. For all results reported in Tables 1 and 2, fused representations from the pretrained dual-path encoder were used to train a Random Forest classifier (200 trees, max depth = 12, max features = sqrt, oob score = True) for final real–fake classification.

Implementation environment. All experiments were implemented in Python 3.8.18 with PyTorch 2.5.1 and CUDA 11.8, using Diffusers 0.33.1, Transformers 4.51.3, and Scikit-learn 1.6.1. Experiments were conducted on an NVIDIA RTX 4090 GPU (24 GB).

### Datasets and evaluation protocol

Ceramic micrograph dataset (CMD): CMD is a custom dataset constructed for this study, containing class-balanced real ceramic micrographs acquired by optical microscopy and fake micrographs generated using a StyleGAN2-ADA^26^-based pipeline. The dataset comprises a total of 1742 images, split into a training set of 1071 images, a validation set of 471 images, and a test set of 200 images, with equal numbers of real and fake samples in each split. Since CMD is a custom dataset constructed in this study, it does not have an external dataset citation and is available from the corresponding author upon reasonable request.

Synthetic ceramic micrographs were generated using StyleGAN2-ADA with the official NVLabs PyTorch implementation. To ensure data integrity, the training, validation, and test splits of the CMD dataset were established prior to StyleGAN2-ADA training, and no real images from the test split were used in GAN training. The generator was trained on real ceramic optical microscopy images resized to 512*×*512 pixels for a total of 1000 kimg, using a batch size of 16, an R1 regularization coefficient of *γ = 8.2*, and adaptive discriminator augmentation (ADA) with an augmentation pipeline of bgc and a target augmentation probability of 0.6. Horizontal mirroring was applied as additional data augmentation. Synthetic images were sampled with a truncation parameter of *ψ = 0.7* to balance generation quality and diversity. The resulting fake micrographs were used as the negative class in the downstream real/fake authenticity classification task.

CIFake dataset: To evaluate cross-domain generalization, FusionDiff was further tested on the CIFake dataset, which contains 120,000 natural images (60k real, 60k fake) with substantially different visual statistics from ceramic micrographs.

Preprocessing: Input images were resized to 512 *×* 512 and normalized using mean and standard deviation of 0.5 across all channels.

## Data Availability

The Ceramic Microstructure Dataset (CMD) generated during the current study is available from the corresponding author upon reasonable request. The CIFAKE dataset used for cross-dataset evaluation is publicly available from its official source at https://www.kaggle.com/datasets/birdy654/cifake-real-and-ai-generated-synthetic-images.

## References

[1] Hirabayashi, Y. et al. Deep learning for three-dimensional segmentation of electron microscopy images of complex ceramic materials. *NPJ Comput. Mater.* **10**(1), 46. 10.1038/s41524-024-01226-5 (2024).

[2] Tang, J. Machine-learning-based, online estimation of ceramic’s microstructure upon the laser spot brightness during laser sintering. *Eng. Sci.* **22**. 10.21203/rs.3.rs-215283/v1 (2023).

[3] Karras, T. et al. Analyzing and improving the image quality of stylegan. In *Proceedings of the IEEE/CVF Conference on Computer Vision and Pattern Recognition*, 8110–8119. 10.1109/cvpr42600.2020.00813 (2020).

[4] Ho, J., Jain, A. & Abbeel, P. Denoising diffusion probabilistic models. *Adv. Neural. Inf. Process. Syst.* **33**, 6840–6851. 10.48550/arXiv.2006.11239 (2020).

[5] Rombach, R., Blattmann, A., Lorenz, D., Esser, P. & Ommer, B. High-resolution image synthesis with latent diffusion models. In *Proceedings of the IEEE/CVF Conference on Computer Vision and Pattern Recognition*, 10684–10695. 10.48550/arXiv.2112.10752 (2022).

[6] Hütten, N. et al. Deep learning for automated visual inspection in manufacturing and maintenance: A survey of open-access papers. *Appl. Syst. Innov.* **7**(1), 11. 10.3390/asi7010011 (2024).

[7] Zajec, P. Few-shot learning for defect detection in manufacturing. *Int. J. Prod. Res.* **62**(19), 6979–6998. 10.1080/00207543.2024.2316279 (2024).

[8] Finn, C., Abbeel, P.& Levine, S. Model-agnostic meta-learning for fast adaptation of deep networks. In *International Conference on Machine Learning*, 1126–1135. 10.48550/arXiv.1703.03400 (PMLR, 2017).

[9] Vinyals, O., Blundell, C., Lillicrap, T. & Wierstra, D. et al. Matching networks for one shot learning. *Adv. Neural Inf. Process. Syst.* **29**. 10.48550/arXiv.1606.04080 (2016).

[10] Snell, J., Swersky, K. & Zemel, R. Prototypical networks for few-shot learning. *Adv. Neural Inf. Process. Syst.* **30**. 10.48550/arXiv.1703.05175 (2017).

[11] Luo, G., Dunlap, L., Park, D. H., Holynski, A. & Darrell, T. Diffusion hyperfeatures: Searching through time and space for semantic correspondence. *Adv. Neural. Inf. Process. Syst.* **36**, 47500–47510. 10.48550/arXiv.2305.14334 (2023).

[12] Ouyang, Y., Xie, L., Zha, H. & Cheng, G. Transfer learning for diffusion models. *Adv. Neural. Inf. Process. Syst.* **37**, 136962–136989. 10.52202/079017-4352 (2024).

[13] He, K., Zhang, X., Ren, S. & Sun, J. Deep residual learning for image recognition. In *Proceedings of the IEEE Conference on Computer Vision and Pattern Recognition*, 770–778. 10.48550/arXiv.1512.03385 (2016).

[14] Dosovitskiy, A., et al. An image is worth 16x16 words: Transformers for image recognition at scale. 10.48550/arXiv.2010.11929 (2020).

[15] Touvron, H., et al. Training data-efficient image transformers & distillation through attention. In *International Conference on Machine Learning*, 10347–10357. 10.48550/arXiv.2012.12877 (PMLR, 2021).

[16] Croitoru, F.-A., Hondru, V., Ionescu, R. T. & Shah, M. Diffusion models in vision: A survey. *IEEE Trans. Pattern Anal. Mach. Intell.* **45**(9), 10850–10869. 10.1109/TPAMI.2023.3261988 (2023). 37030794

[17] Yang, J., Wan, H. & Shang, Z. Enhanced hybrid CNN and transformer network for remote sensing image change detection. *Sci. Rep.* **15**(1), 10161. 10.1038/s41598-025-94544-7 (2025). 40128281 PMC11933460

[18] Huang, G., Liu, Z., Van Der Maaten, L. & Weinberger, K.Q. Densely connected convolutional networks. In *Proceedings of the IEEE Conference on Computer Vision and Pattern Recognition*, 4700–4708. 10.48550/arXiv.2006.11239 (2017).

[19] Liu, Z., et al. Swin transformer: Hierarchical vision transformer using shifted windows. In *Proceedings of the IEEE/CVF International Conference on Computer Vision*, 10012–10022. 10.1109/iccv48922.2021.00986 (2021).

[20] Radford, A., et al. Learning transferable visual models from natural language supervision. In *International Conference on Machine Learning*, 8748–8763. 10.48550/arXiv.2103.00020 (PMLR, 2021).

[21] Kaddes, M. et al. Breast cancer classification based on hybrid CNN with LSTM model. *Sci. Rep.* **15**(1), 4409. 10.1038/s41598-025-88459-6 (2025). 39910136 PMC11799331

[22] Chen, T., Kornblith, S., Norouzi, M. & Hinton, G. A simple framework for contrastive learning of visual representations. In *International Conference on Machine Learning*, 1597–1607. 10.48550/arXiv.2002.05709 (2020).

[23] Bird, J. J. & Lotfi, A. Cifake: Image classification and explainable identification of ai-generated synthetic images. *IEEE Access* **12**, 15642–15650. 10.1109/access.2024.3356122 (2024).

[24] Kingma, D.P. & Welling, M. Auto-encoding variational bayes. arXiv preprint. 10.48550/arXiv.1312.6114 (2013).

[25] Hu, E.J., et al. LoRA: Low-rank adaptation of large language models. In *International Conference on Learning Representations*. 10.48550/arXiv.2106.09685 (2022).

[26] Karras, T. et al. Training generative adversarial networks with limited data. *Adv. Neural. Inf. Process. Syst.* **33**, 12104–12114. 10.48550/arXiv.2006.06676 (2020).

